# Menstrual and reproductive outcomes after use of balloon tamponade for severe postpartum hemorrhage

**DOI:** 10.1186/s12884-018-2085-6

**Published:** 2018-11-21

**Authors:** Choi Wah KONG, William Wing Kee TO

**Affiliations:** Department of Obstetrics and Gynaecology, United Christian Hospital, 130 Hip Wo Street, Kwun Tong, Hong Kong

**Keywords:** Intrauterine balloon tamponade, Fertility, Menstruation, Postpartum hemorrhage, Pregnancy

## Abstract

**Background:**

The use of intrauterine balloon tamponade to manage postpartum hemorrhage is increasing. However, there is lack of studies on the menstrual and reproductive outcomes after such treatment. The purpose of this study is to explore the menstrual and reproductive outcomes for patients who had been managed by intrauterine balloon tamponade for severe postpartum hemorrhage in her index pregnancy.

**Methods:**

All patients who had delivered in United Christian Hospital from January 2011 to June 2016 with severe postpartum hemorrhage (PPH) (blood loss> = 1 L) were identified by the labour ward delivery registry and a comprehensive obstetric database. Patients who had intrauterine balloon tamponade inserted were compared with those managed solely by uterotonic agents as controls. Patients who had hysterectomy or additional procedures performed, such as compression sutures or uterine artery embolization were excluded from both groups. A questionnaire on menses, fertility and reproductive outcomes was mailed to both groups of patients. Those that had not replied within 4 weeks would receive a telephone survey.

**Results:**

A total of 39 patients in the balloon tamponade group and 161 patients in the control group were recruited, which represented 87.0% of all eligible patients within the study period. The median follow up period was 45 months. All patients in the balloon tamponade group had return of menses after delivery. The majority of the patients (87.2%) in the balloon tamponade group had normal menstrual patterns in the 12 months after the index delivery as well as in the most recent 12 months. After excluding the patients with contraception, the subsequent pregnancy rate was 42.9% (9/21) in the balloon tamponade group compared to 45.9% (28/61) in the control group (*p* = 0.81). Among the 9 subsequent pregnancies in the balloon tamponade group, there were two miscarriages, one scar pregnancy, one induced abortion, while the remaining five were normal pregnancies with full term deliveries without intrauterine growth restriction. The majority of patients replied that they were satisfied with using Bakri balloon for PPH management in their index pregnancy.

**Conclusions:**

Intrauterine balloon tamponade for the management of severe PPH appeared to pose little adverse effects on subsequent menstrual and reproductive function.

**Electronic supplementary material:**

The online version of this article (10.1186/s12884-018-2085-6) contains supplementary material, which is available to authorized users.

## Background

The incidence of postpartum hemorrhage is around 5% and is continuously increasing worldwide due to increase in patients with advanced maternal age, multiple pregnancies, caesarean deliveries and previous caesarean sections with abnormal placentation [1–4]. Using intrauterine balloon to manage severe postpartum hemorrhage (PPH) is shown to be effective and can decrease the rate of hysterectomy [5–8]. As a result, intrauterine balloon has been more and more commonly incorporated into severe PPH protocols. It has become an integral part of the “HEMOSTASIS” management algorithm advocated in the United Kingdom [9]. However, follow-up studies on the menstrual and reproductive outcomes after the use of balloon tamponade are minimal. There are only two studies in the literature that has specifically reviewed patients’ menses after the use of balloon tamponade, of which one was a case series that included only 5 patients [10] while the other included 33 women but was only published as electronic poster [11]. For the reproductive outcomes, there are only five studies available that have reported a total of 14 patients’ subsequent pregnancy and delivery after the use of balloon tamponade [11–14]. However, the details on subsequent pregnancy outcome such as the gestational age at delivery were not reported in at least half of the cases. Therefore this study aims at reviewing an unselected cohort of patients who had been managed with intrauterine balloon for severe PPH in their index pregnancy over a period of 66 months. Using this larger sample size, we attempted to evaluate the details of the patients’ menstrual functions, fertility and reproductive outcomes, in order to verify the long term impact of balloon tamponade on these patients with severe postpartum hemorrhage in their previous pregnancies.

## Methods

This was a retrospective cohort study. All pregnant patients with deliveries in United Christian Hospital from January 2011 to June 2016 who had severe PPH (blood loss > = 1 L) were recruited. These patients were identified from our previous studies [15, 16] and their demographic and clinical data were reviewed. Patients who had uterine balloon inserted were compared with those severe PPH patients who were managed solely by uterotonic agents which served as the control group.

The department protocol of management of severe postpartum hemorrhage was attached in Additional file 1. The Bakri intrauterine balloon catheter (Cook Medical, Bloomington [IN], US) was the only available balloon tamponade for this purpose in our department. The procedure for application of the Bakri balloon was in accordance with that generally described in the literature [5–7]. When the balloon was inserted during a caesarean section, the distal end of the balloon shaft was passed through the cervical opening with an assistant pulling the end per vagina. The amount of saline instilled was the amount considered sufficient by the operator to produce a “positive tamponade test” [5, 9], up to maximum of 500 ml. If bleeding was arrested after balloon inflation, the balloon tamponade was removed within 24 h after insertion.

For patients who had intrauterine balloon tamponade attempted or inserted, if they had hysterectomies or additional procedures performed such as compression sutures and uterine artery embolization (UAE), they would be excluded in both the balloon tamponade group and the control group as these additional procedures themselves may affect the menstrual and reproductive outcomes.

A questionnaire on menstrual patterns, fertility and reproductive outcomes was mailed to both groups of patients (see Additional files 2 and 3). A consent form was attached to the questionnaire and written consent was obtained. The questionnaire and consent form were designed in two languages: traditional Chinese and English. They were mailed to the patient with language appropriate to her need. Patients were asked to return the completed questionnaire and consent form by mail with a pre-paid return envelope or by fax to a specific line to the principal investigator. If no reply was received from the patient after 4 weeks, the principal investigator would attempt to contact the patient by phone to conduct a telephone survey and the same questions as in the mailed questionnaire would be asked. Patients would also be contacted by phone by the principal investigator to clarify any queries in their questionnaire reply if deemed necessary. Additional data was collected from the patients involved in the previous studies, for the current study. Ethics approval for this study was granted by the Kowloon Central/ Kowloon East Ethics Committee Board of the Hospital Authority, Hong Kong. (KC/KE-16-0169/ER-1).

The primary outcome was the percentage of patients that had normal menses after delivery while the secondary outcomes were the percentage of patients that had subsequent pregnancies. SPSS for Windows package (SSPS Inc., Chicago, IL Version 23.0) was used for data entry and analysis. The differences between continuous variables were analyzed using student’s t-test. The differences between discrete variables were analyzed by Chi-square test or Fisher’s exact test when appropriate. A *p*-value of < 0.05 was considered statistically significant.

## Results

**Fig. 1:**
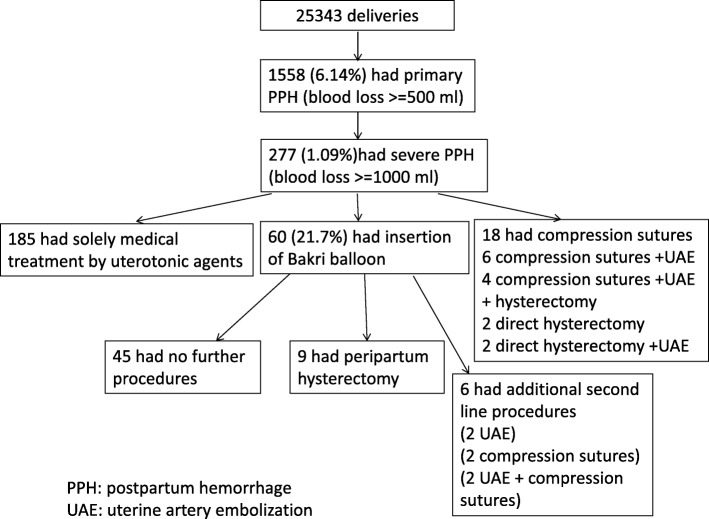
Number of patients with various treatments for severe postpartum hemorrhage

There were a total of 25,343 deliveries during the study period. The frequency of primary PPH with an estimated blood loss exceeding 500 mL was 6.15% (*n* = 1558). The frequency of massive PPH with an estimated blood loss exceeding 1 L was 1.09% (*n* = 277). Among these patients, Bakri balloon tamponade was attempted in 60 (21.7%) of them, among which, there was 9 cases with peripartum hysterectomy performed and in which one case had maternal death. Balloon tamponade was successful in arresting PPH without hysterectomy in 51 out of 60 patients (85%). In these 51 patients with balloon tamponade inserted, 6 patients required additional procedures including UAE (*n* = 2), compression sutures (n = 2) and both (n = 2). A total of 185 patients had severe PPH that were successfully managed solely with uterotonics served as the control group (Fig. 1).

Among the 45 patients in the balloon tamponade group, 39 completed the questionnaire survey giving a response rate of 86.7%. Within the control group, 161 patients completed the questionnaire survey giving a comparable response rate of 87.0%. The overall recruitment rate was 200/230 (87.0%), of which 141 patients replied to the written questionnaire and 59 patients replied to the telephone survey. None of the patients that could be contacted refused to participate in the survey. There were no significant differences in the basic epidemiological characteristics or clinical history between the recruited subjects and those patients that could not be contacted.

The mean interval between the survey and the index pregnancy was 47.7 (SD 16.5) months for the balloon tamponade group and 44.1 (SD 17.0) months for the control group. There were no significant differences in basic epidemiological parameters including the maternal age, parity, mode of delivery, or the aetiology of the severe PPH between the two groups of patients. The balloon tamponade group had significantly more blood loss and higher incidence of blood product transfusion than the control group. There was no difference in the incidence of postpartum endometritis between the two groups (Table 1).

**Table 1:** Clinical parameters between Bakri balloon group vs control group

	Bakri balloon only (*n* = 39)	Control (*n* = 161)	*p*-value; MD; (95% CI)
Maternal age (years)	33.9 (4.00)	34.6 (4.98)	0.442; − 0.66 (−2.35 to 1.03)
Gestation at delivery (weeks)	38.1 (2.07)	38.3 (2.43)	0.78; −0.12 (− 0.95 to 0.71)
Parity			
Primiparous	19 (48.7%)	71 (44.1%)	0.60
Multiparous	20 (51.3%)	90 (55.9%)	
Multiple pregnancy	7 (17.9%)	17 (10.6%)	0.27
Previous caesarean section	5 (12.8%)	52 (32.3%)	*0.02
Mode of delivery			
Normal vaginal	9 (23.1%)	27 (16.8%)	0.56
Instrumental	1 (2.6%)	8 (5.0%)	
Caesarean section	29 (74.3%)	126 (78.3%)	
Cause of severe PPH			
Uterine atony	24 (61.5%)	115 (71.4%)	0.38
Placenta praevia/accreta	12 (30.8%)	33 (20.5%)	
Genital tract trauma	3 (7.7%)	13 (8.1%)	
Total blood loss (ml)	1812 (748)	1440 (413)	*< 0.001; 371 (197 to 545)
Blood product transfusion	35 (89.7%)	91 (56.5%)	*< 0.001
Coagulopathy	11 (28.2%)	34 (21.1%)	0.34
Presence of endometritis after delivery	1 (2.6%)	3 (1.9%)	0.583

*MD* Mean difference, *CI* Confidence interval

*statistically significant

One patient in the control group was diagnosed to have polycystic ovarian syndrome with prolonged periods of amenorrhoea prior to her index pregnancy, which was induced by in-vitro fertilization. She reported secondary amenorrhoea for over 4 years up to the time of the survey, though she has been breast feeding for over 20 months in the postpartum period. Her prolonged amenorrhoea was ascribed to her chronic anovulation similar to her pre-pregnancy state. When the menstrual patterns were compared between the two groups, it could be seen that after excluding the patient with permanent amenorrhoea, the menstrual pattern of both groups of patients were largely normal in the 12 months after the index delivery as well as in the most recent 12 months. (Tables 2 and 3).

**Table 2:** Menstrual pattern within 12 months postpartum between Bakri balloon group vs control group

	Bakri balloon only (*n* = 39)	Control (*n* = 161)	p-value; MD; (95% CI)
Duration of breastfeeding postpartum (months)	4.41 (6.05); median 1.5 range 0–24 m	3.25 (4.44); median 2.0 range 0–30 m	0.176; 1.16 (− 0.52 to 2.84)
Return of menses after delivery (months)	4.43 (4.68)	4.95 (3.24)	0.42; −0.51 (−1.77 to 0.74)
Menstrual pattern within 12 months postpartum			
No return of menses	0	1^†^	1.00
Cycle regularity			0.47
Regular 28 (±7) day cycles	34 (87.2%)	141 (88.1%)	
Short (< 21 day) or long (> 35 day) cycles	3 (7.7%)	16 (10.0%)	
Totally irregular cycles	2 (5.1%)	3 (1.9%)	
Duration of flow			0.41
Little flow < 2 days	0 (0.0%)	3 (1.9%)	
Normal flow (3–7 days)	35 (89.7%)	148 (92.5%)	
Prolonged flow (> 7 days)	4 (10.3%)	9 (5.6%)	
Amount of flow			0.172
Heavy	6 (15.4%)	23 (14.4%)	
Normal	26 (66.7%)	124 (77.5%)	
Scanty	7 (17.9%)	13 (8.1%)	
Presence of dysmenorrhoea			0.46
No dysmenorrhoea	31 (79.5%)	136 (85.0%)	
Mild	8 (20.5%)	22 (13.8%)	
Severe requiring medication	0 (0.0%)	2 (1.2%)	

*MD* Mean difference, *CI* Confidence interval

†One patient with prolong secondary amenorrhoea was excluded from subsequent analysis

**Table 3:** Menstrual pattern in recent 12 months postpartum between Bakri balloon group vs control group

	Bakri balloon only (n = 39)	Control (n = 161)	p-value; MD (95% CI)
Mean interval from delivery to survey (months) (SD)	47.7 (16.5)	44.1 (17.0)	0.26; 3.44 (−2.52 to 9.40)
Cycle regularity			0.20
No menses	0	1^†^	
Regular 28 (±7) day cycles	34 (87.2%)	127 (79.4%)	
Short (< 21 day) or long (> 35 day) cycles	2 (5.1%)	25 (15.6%)	
Totally irregular cycles	3 (7.7%)	8 (5.0%)	
Duration of flow			0.95
Little flow < 2 days	0 (0.0%)	6 (3.7%)	
Normal flow (3–7 days)	34 (87.2%)	143 (89.4%)	
Prolonged flow (> 7 days)	5 (12.8%)	11 (6.9%)	
Amount of flow			0.46
Heavy	6 (15.4%)	28 (17.5%)	
Normal	27 (69.2%)	118 (73.8%)	
Scanty	6 (15.4%)	14 (8.7%)	
Presence of dysmenorrhoea			0.36
No dysmenorrhoea	31 (79.5%)	130 (81.2%)	
Mild	8 (20.5%)	24 (15.0%)	
Severe requiring medication	0 (0.0%)	6 (3.8%)	

*MD* Mean difference, *CI* Confidence interval, *SD* Standard deviation

†One patient with prolong secondary amenorrhoea was excluded from subsequent analysis

Concerning the reproductive outcomes, 46.2% (18/39) of the patients in the balloon tamponade group practised contraception after the index pregnancy compared with 62.1% (100/161) of the patients in the control group. After excluding those with contraception, nine patients (42.9%) in the balloon tamponade group had subsequent pregnancies, of which 5 were live births, 2 were miscarriages, 1 was a scar ectopic pregnancy and 1 had termination of pregnancy due to social reasons. For the 5 livebirths, all were term live births without intrauterine growth restriction and 2 patients had PPH again in their subsequent pregnancies but both were managed successfully with oxytocin. Within the control group, 28 patients (45.9%) had subsequent pregnancies, of which 16 were live births, 5 were miscarriages, 2 had ectopic pregnancies with one of them being a scar ectopic, and 5 had termination of pregnancy. For the 16 livebirths, 2 were born preterm at 31 and 36 week of gestation and 6 patients had PPH again in their subsequent pregnancies which were managed by oxytocin. There were no significant differences between the subsequent pregnancy rate and live birth rate between the Bakri balloon and control group (*p* = 0.81 and *p* = 0.83). (Table 4).

**Table 4:** Reproductive outcomes between Bakri balloon group vs control group

	Bakri balloon only (n = 39) (SD)	Control (n = 161) (SD)	p-value; MD; 95% CI
Use of contraception	18 (46.2%)	100 (62.1%)	0.07
Subsequent pregnancies#	9 (42.9%)	28 (45.9%)	0.81
Occurrence after index delivery†(months)	28.4 (13.0); range 11–48	28.6 (10.9); range 10–52	0.98; −0.13; −8.99 to 8.74
Miscarriage	2 (9.5%)	5 (8.2%)	1.00
Termination	1 (4.8%)	5 (8.2%)	1.00
Ectopic	1 (4.8%)	2 (3.3%)	1.00
Deliveries	5 (23.8%)	16 (26.2%)	0.83
Vaginal delivery	4	11	
Caesarean section	1	6	
Presence of PPH	2	6	

*SD* Standard deviation, *MD* Mean difference, *CI* Confidence interval

# The % of subsequent pregnancies was calculated by excluding those patients that had contraception

†The subsequent pregnancy outcome of each patient is counted on their first subsequent pregnancy

The maternal satisfaction rate with the use of balloon tamponade was 37/39 (94.9%). Only 2 patients expressed dissatisfaction with the balloon tamponade treatment in the survey. One of them had secondary PPH requiring surgical evacuation yielding decidualized tissue compatible with postpartum endometritis. Despite a full course of antibiotic treatment, the patient continued to have prolonged lochia which lasted for up to 3 months post-delivery. In the other patient, she was dissatisfied with Bakri balloon treatment due to pain experienced during the insertion of the balloon.

## Discussion

The current survey of patients with Bakri balloon insertion for severe PPH in their index pregnancies confirmed that the majority of them would have normal menstrual patterns and reproductive performance.

PPH continues to be one of the world’s leading causes of maternal mortality [17]. Traditionally, peripartum hysterectomy would be performed in patients with massive PPH as a life-saving rescue procedure in those who failed to respond to uterotonics. However, due to the high associated morbidity associated with peripartum hysterectomy, various conservative surgical procedures have been developed to reduce the need for hysterectomy, including external compression sutures, selective devascularization by surgical ligation or radiological embolization of the uterine and pelvic arteries [5, 18–20]. However, most of these studies focused on the success rates in avoiding hysterectomy, with little direct data available on the actual preservation of fertility in these women, including the return of menses, the pregnancy rate, or the outcome of subsequent pregnancies. In addition, further limitations to gathering such data could result from the possible bias arising from the reluctance of PPH patients to pursue a subsequent pregnancy [21].

Of the 21 successful pregnancies in our cohort, 8 were reported to have PPH controlled by oxytoxics. From a large North American cohort, it was shown that women with a history of PPH had a 3-fold increased risk of PPH in their second pregnancy (15%) compared with unaffected women (5.0%); and in a third pregnancy, the risk rose to 26.6% after 2 previously affected pregnancies. In addition, it was observed that recurrent PPH in subsequent pregnancies was particularly higher in those with a previous severe PPH. Such observations were compatible with what was observed in our survey [22]. In a case series that studied pregnancy outcome in women who had radiological pelvic arterial embolization for postpartum hemorrhage, it was also shown that there was a significantly higher rate of placenta accreta as compared to women without embolization in their previous pregnancy (23.5% vs 0%). This high incidence of recurrent PPH was probably more related to predisposing risk factors that had existed even for the index pregnancy [23]. Thus, regardless of the technique used in the management of PPH, the risk of recurrent PPH in subsequent pregnancies is substantial [21].

So far, there were more studies in the literature targeted at reviewing menstrual and fertility outcomes after pelvic vascular ligation, radiological embolization and compression sutures for PPH than after balloon tamponade. For instance, a relatively large cohort of 68 patients with hypogastric artery ligation for PPH reported no increase in subsequent subfertility when compared to controls [24], and another series of 53 women who had uterine artery embolization also showed no statistically significant differences of occurrence of pregnancy between the embolized and non-embolized groups [25]. Uterine compression sutures have been shown to be associated with subsequent uterine synechiae formation as demonstrated by hysteroscopy in up to 26% of the cases, though the impact of this on pregnancy outcome is uncertain [26]. However, such synechiae formation has not been reported to be associated with balloon tamponade so far. A recent review has raised the possible increased risks of endometritis in those with balloon management [27]. In our cohort, all the Bakri balloons were removed within 24 h after insertion and there was no significantly increase in this incidence after Bakri balloon insertion. Therefore, Bakri balloon appears to be safe and has no long term effect on subsequent menstrual and reproductive function. A systematic review on the fertility rates and subsequent pregnancy outcome after conservative surgical procedures for PPH has reported that compressive sutures were associated with good pregnancy outcomes but an increasing rate of subsequent repeat caesarean section, while pelvic artery embolization is more likely to be associated with subsequent placental disorders, leading to fetal growth restriction, preeclampsia, and placenta accreta and/or praevia [21].

There were only two studies that had assessed the menstrual function after balloon tamponade. In a small series of five patients who underwent balloon tamponade procedure for severe PPH, all of them had return of menses within 8 week after delivery or after cessation of breast feeding, with normal amount and duration of menses [10]. Another larger cohort of 33 women reported 81.8% had regular menses after balloon tamponade insertion, though this study was only published as electronic poster [11]. There were also only scanty reports of pregnancy outcome after balloon tamponade insertion. The pregnancy outcome of the above small series of five patients reported successful term pregnancies in all of them, though one of them had recurrent PPH controlled by oxytoxics [10]. The authors concluded that the uterine-specific Bakri balloon should pose minimal effect on menses, fertility and future pregnancies. In another French series of 49 women who had intrauterine balloon tamponade with an overall 65% success rate, it was reported that two women had a subsequent full-term pregnancy without recurrence of postpartum hemorrhage [14]. In another report of 2 cases using the Roush balloon, one woman was reported to have a subsequent uncomplicated pregnancy but the details were not given [12]. Moreover, among 5 patients who underwent uterine sandwich technique with compression sutures followed by Bakri balloon insertion for uterine atony, it was reported that 2 of the women had subsequent successful pregnancies, though one of them had recurrent PPH due to uterine atony [13]. In the cohort of 33 women as mentioned above, it was reported that 4 patients (19.0%) with subsequent pregnancy and all the deliveries were uncomplicated [11]. Although not all the clinical details of the subsequent pregnancies were available in all of the above studies, their reports supported our findings that the use of intrauterine balloon tamponade did not affect subsequent menstrual and reproductive outcomes.

For other complications of Balloon tamponade, Franklin-Dumont et al. compared 13 patients with Bakri balloon inserted for postpartum hemorrhage with 351 patients without Bakri balloon inserted and found significantly increased in rate of endometritis after the use of Bakri balloon [27]. In our cohort, all the Bakri balloons were removed within 24 h after insertion and there was no significantly increase in the incidence of endometritis after Bakri balloon insertion. Therefore, Bakri balloon appears to be safe and has no long term effect on subsequent menstrual and reproductive function.

The strength of our current study was its focus on reviewing specifically the menstrual pattern and subsequent pregnancy outcome of this target group, and our data have comprehensively described the reproductive performance after specifically excluding involuntary subfertility in this cohort, instead of merely reporting the number of pregnancies as they occurred. Apparently, our cohort had reported the largest number of subsequent pregnancies reported so far in the literature. The median follow up time was around 45 months, which should be adequate to demonstrate any long term impact of the previous balloon procedure on menstrual or reproductive function.

The limitations of this study included the risk of selection bias in this cohort as the characteristic of the group of women with Bakri balloon was somewhat different from the control group, with higher blood loss and a higher incidence of blood product transfusions. However, while the higher blood loss was obviously the indication for balloon tamponade management in the first place, even with this potential confounding factor, the women in the balloon group did not have poorer menstrual and fertility outcome compared with the control group, implying that intrauterine balloon management should pose no effects on these outcomes. Another potential weakness of this cohort was the inability to assess the natural timing for return of menses after the procedure, as in many of the women, the return of menses was delayed due to breast feeding. There may also be recall bias for the menstrual outcome as the mean time interval from delivery to the survey was close to 4 years but the recall bias for subsequent pregnancy outcome should be minimal. Finally, a larger cohort would be needed to evaluate the risks of recurrent PPH or other obstetric complications in subsequent pregnancies.

## Conclusions

Intrauterine balloon tamponade for the management of severe PPH appeared to pose little adverse effects on subsequent menstrual and reproductive function. The pregnancy outcome was similar to those managed conservatively with uterotonic agents alone. Intrauterine balloon tamponade should be recommended to be used as second line treatment for severe postpartum hemorrhage.

## Additional files

Additional file 1: Department protocol for severe PPH. This file is the department protocol for severe postpartum hemorrhage in United Christian Hospital. (DOCX 21 kb)

Additional file 2: Questionnaire on menstrual patterns, fertility and reproductive outcomes after severe postpartum hemorrhage (balloon group). This file is the questionnaire that mailed to the patients with evere postpartum hemorrhage that were managed by balloon tamponade. (DOCX 30 kb)

Additional file 3: Questionnaire on menstrual patterns, fertility and reproductive outcomes after severe postpartum hemorrhage (control group). This file is the questionnaire that mailed to the patients with severe postpartum hemorrhage that were solely managed by uterotonic agents. (DOCX 29 kb)

Additional file 4: Dataset. This file is the dataset of this study. (XLSX 34 kb)

## Abbreviation

PPH: Postpartum hemorrhage
