# Supplementary material for: Menstrual and reproductive outcomes after use of balloon tamponade for severe postpartum hemorrhage
Source: BMC Pregnancy Childbirth. 2018 Nov 21;18:451. doi: 10.1186/s12884-018-2085-6 (PMC6249747; doi:10.1186/s12884-018-2085-6)
Supplement: Supplementary file 1 — Department protocol for severe PPH. This file is the department protocol for severe postpartum hemorrhage in United Christian Hospital. (DOCX 21 kb) [file 12884_2018_2085_MOESM1_ESM.docx]

## Department protocol for severe PPH

## Severe PPH (>=1000 ml)

1. Call for help
   - 3^rd^ call should be informed if blood loss >=1500 ml. Consultant should be informed if blood loss >=3000 ml.
   - Alert anesthetist +/- ICU doctor after assessment by 3^rd^ call
     - when the patient failed to respond to first line resuscitation with persistent haemodynamic instability e.g. SBP <90mmHg and HR >120/min.
     - For support on difficult vascular access not established during vigourous resuscitation.
   - Alert blood bank for the need of massive transfusion.
2. Resuscitation of patient

- Two 16-gauge IV line, consider CVP if necessary
- Replace IV fluid at fast rates from pre-warmed fluid stock. Replace with crystalloid first max up to 2L, followed by Colloids (Max 1.5L) if necessary.
- Initiate blood transfusion early. Blood (matched or unmatched) to be given if hypotension persists after 1000 ml of crystalloid or colloid infusion. Use warming coil as appropriate.
- Cross match 4 units of whole blood.
- Give FFP 4 units per 6 units of blood or if PT/APTT > 1.5 times of normal.
- Give platelet concentrate if platelet count < 50 x 109/L.
- To reduce hypothermia by either warming up the delivery suite to 24 degree Celsius or cover the patient with warming blanket. Measure the temperature of the patient hourly.

1. Find and treat the cause
   - Uterine atony
     - Uterine massage or apply bimanual compression
     - Give syntocinon 5 units or Syntometrine 1ampoule IV
     - Give 30 unit syntocinon infusion in 500 ml NS (rate 90-160 ml/hr)
     - Carboprost
       - 0.25 mg IM, can be repeated every 15 min up to a max of 8 doses
       - direct intramyometrial injection of carboprost 0.5 mg can be given (off-label use)
       - Use with caution in patients with asthma, severe hypertension, active cardiac/ pulmonary, renal or hepatic diseases.
       - 3rd call should be informed if bleeding was not controlled after 2 doses of carboprost
     - rectal misoprostol 1000μg
   - Retained placenta
     - The placenta is examined for completeness. If suspected incomplete placenta, the uterus should be explored as described under section "Exploration Of Uterus".
   - Genital tract trauma
     - The perineum and the cervix should be inspected and any bleeding tear should be repaired. The repair may need to be performed under anesthesia in operation theater.
2. Monitoring of patient
   - Insert Foley catheter and check urine output hourly
   - Measure BP/Pulse every 15 minutes and apply continuous pulse oximetry. Measure BP/Pulse every 5 minutes if patient is haemodynamically unstable.
   - Apply cardiac monitoring if pulse >=120 or if blood loss >=1500 ml
   - Blood Tests: CBP, LRFT, coagulation profile ± blood gases, check fibrinogen level if blood loss > =2L. The need and frequency of Haemocue to be ordered by attending obstetricians/anaethestists.
   - Save all swabs, linens and pads for blood loss estimation.
3. If medical measures fails to control bleeding, one or more of the following second line measures can be considered and 3^rd^ call should be informed:

- Bakri balloon
  - - Bakri balloon can be either inserted vaginally or abdominally through uterine incision.
    - No more than 500 ml of saline should be infused into the Bakri balloon.
    - The balloon output should be monitored every 15 min for the first 2 hours. A cumulative charting of the drain output is advised for the first two hours.
    - The balloon should be removed at no more than 24 hours after insertion.
    - Vaginal packing can be applied if there is slipping out of the balloon from the cervical os.
    - Traction may be applied to the Bakri balloon for compression onto lower placental bed.
- Compression sutures
  - - Compression sutures can be applied during caesarean sections; in case of vaginal delivery, laparotomy will be needed in order to perform compression sutures.
- Uterine artery embolization
  - - Contact radiologist for arrangement and the patient should be escorted by an obstetrician +/- midwife for transfer to the X-ray department and during the procedure.

1. If patient is needed to be transferred to operation theater to control bleeding, consider requesting anaethestist to escort patient if patient is haemodynmatically unstable. Resuscitation bag should be prepared and transferred together with patient. Details please refer to the guidelines on transport of critically ill parturients.
2. If second line measures fail to control bleeding or if patient is hemodynamically unstable with rapid massive blood loss, pelvic devascularization and/or hysterectomy should be performed with consultant involvement.
