# Supplementary material for: Menstrual and reproductive outcomes after use of balloon tamponade for severe postpartum hemorrhage
Source: BMC Pregnancy Childbirth. 2018 Nov 21;18:451. doi: 10.1186/s12884-018-2085-6 (PMC6249747; doi:10.1186/s12884-018-2085-6)
Supplement: Supplementary file 3 — Questionnaire on menstrual patterns, fertility and reproductive outcomes after severe postpartum hemorrhage (control group). This file is the questionnaire that mailed to the patients with severe postpartum hemorrhage that were solely managed by uterotonic agents. (DOCX 29 kb) [file 12884_2018_2085_MOESM3_ESM.docx]

Hospital Authority Kowloon East Cluster:

Department of Obstetrics & Gynaecology of United Christian Hospital

Topic: Menstrual and reproductive outcome after severe postpartum haemorrhage

Objective: The incidence of postpartum haemorrhage is increasing. However, the menses, fertility and reproductive outcome after postpartum hemorrhage has not been studied in detail. We would like to explore these aspects, and the answers you provide should help us to manage postpartum haemorrhage more effectively and safely.

You had delivered your baby in United Christian Hospital in year _______ with postpartum haemorrhage. Please complete the following questionnaire and tick on the box if applicable. Please mail back to us by the attached return envelope or by fax. We may contact you by telephone for more information if necessary.

1. After the delivery in year _____, did you breast-feed your baby and if yes, what is the duration of the breast feeding?

❑ No ❑ Yes, for _______ months

1. When did your menses return after that delivery?

________ months after that delivery

1. Please describe your menses within 12 months after that delivery?
2. The regularity of the menses

❑ regular menses every month ( around 4 weeks)

❑ irregular menses with cycles shorter than 3 weeks or longer than 6 weeks

❑ totally irregular menses with no fixed pattern

1. The duration of the menses

❑ <= 2 days ❑ 3 -7 days ❑ >=7 days

1. Amount of menses compared to before the pregnancy

❑ heavy flow ❑ normal flow ❑ little flow

1. Any pain during menses?

❑ No ❑ Yes

Require medication to control the pain?

❑ No ❑ Yes

1. Please describe your menses in recent 12 months?
2. The regularity of the menses

❑ regular menses every month ( around 4 weeks)

❑ irregular menses with cycles shorter than 3 weeks or longer than 6 weeks

❑ totally irregular menses with no fixed pattern

1. The duration of the menses

❑ <= 2 days ❑ 3 -7 days ❑ >=7 days

1. Amount of menses

❑ heavy flow ❑ normal flow ❑ little flow

1. Any pain during menses?

❑ No ❑ Yes

Require medication to control the pain?

❑ No ❑ Yes

1. Did you use any contraception after that delivery? When did you start the contraception and how long have you continued contraception? Have you been attempting pregnancy after that delivery but have problems getting pregnant?

❑ No, I did not have contraception after that delivery.

❑ Yes, I started contraception at _____ months after delivery and continued for ________months/ continued till now

❑ Yes, I have been attempting to get pregnant since _____ months after delivery and have not been successful for ________months/ till now

1. Did you have any miscarriage or termination of pregnancy after that delivery? If yes, please specify the number and the time of the miscarriage/termination of pregnancy.

- No, I did not have any miscarriage or termination of pregnancies after that delivery.

❑ Yes, I have _____ miscarriage after that delivery, the miscarriage occurred _____ months after that delivery. (If there is more than one miscarriage, only need to state the time for the earliest miscarriage.)

❑ Yes, I have _____ termination of pregnancy after that delivery, the termination occurred _____ months after that delivery. (If there is more than one termination, only need to state the time for the earliest termination.)

1. Did you deliver any further babies after the delivery in year _____? If yes, please specify the number and the time of the delivery.

- No, I did not deliver any babies after that delivery. (🡪 please go to answer question 9)

❑ Yes, I have _____ further delivery after that delivery, it occurred _____ months after that delivery. (If there is more than one delivery, only need to state the time for the earliest delivery.)

(🡪 please go to answer question 8)

1. We would like to know the details for that new delivery. (If there was more than one delivery after that delivery, please only answer the following questions regarding to the earliest delivery that occurred afterwards)
2. Was it term baby? If it is preterm, please state the gestation at delivery.

❑ Yes

❑ No, it is preterm, delivered at _____ weeks of gestation

1. Were there any antenatal complications for the pregnancy? If yes, please specify the complication such as intrauterine growth restriction.

❑ No

❑ Yes, the complication was: ______________________________________

1. What was the mode of delivery for the pregnancy?

❑ Normal vaginal delivery ❑ Vacuum/forceps delivery ❑ Caesarean section

1. Did you have postpartum haemorrhage during this new delivery? If yes, have you been treated by balloon tamponade for the pregnancy?

❑ No, I did not have postpartum haemorrhage for that pregnancy.

❑ Yes, I had postpartum haemorrage and was treated by balloon tamponade

❑ Yes, I had postpartum haemorrage but was not treated by balloon tamponade

~~~~~~~~~~~~~~~~~~~~~~~~~~~~~~~~End~~~~~~~~~~~~~~~~~~~~~~~~~~~~~~~~~~

Thank you for completing this questionnaire!
